# Functional Connectivity of the Dorsal Striatum in Female Musicians

**DOI:** 10.3389/fnhum.2016.00178

**Published:** 2016-04-22

**Authors:** Shoji Tanaka, Eiji Kirino

**Affiliations:** ^1^Department of Information and Communication Sciences, Sophia UniversityTokyo, Japan; ^2^Department of Psychiatry, Juntendo University School of MedicineTokyo, Japan; ^3^Juntendo Shizuoka HospitalShizuoka, Japan

**Keywords:** connectivity, corticostriatal, graph theory, motor, music, network, procedural learning, skill

## Abstract

The dorsal striatum (caudate/putamen) is a node of the cortico-striato-pallido-thalamo-cortical (CSPTC) motor circuit, which plays a central role in skilled motor learning, a critical feature of musical performance. The dorsal striatum receives input from a large part of the cerebral cortex, forming a hub in the cortical-subcortical network. This study sought to examine how the functional network of the dorsal striatum differs between musicians and nonmusicians. Resting state functional magnetic resonance imaging (fMRI) data were acquired from female university students majoring in music and nonmusic disciplines. The data were subjected to functional connectivity analysis and graph theoretical analysis. The functional connectivity analysis indicated that compared with nonmusicians, musicians had significantly decreased connectivity between the left putamen and bilateral frontal operculum (FO) and between the left caudate nucleus and cerebellum. The graph theoretical analysis of the entire brain revealed that the degrees, which represent the numbers of connections, of the bilateral putamen were significantly lower in musicians than in nonmusicians. In conclusion, compared with nonmusicians, female musicians have a smaller functional network of the dorsal striatum with decreased connectivity. These data are consistent with previous anatomical studies reporting a reduced volume of the dorsal striatum in musicians and ballet dancers, suggesting that long-term musical training reshapes the functional network of the dorsal striatum to be less extensive or selective.

## Introduction

Professional musicians undergo long-term training that includes the acquisition of motor skills required to play a musical instrument. Performing music, either playing a musical instrument or singing, requires complex information processing and therefore activates several brain regions simultaneously. There are numerous tasks that can be used to measure brain activity during music-related processing. In a positron emission tomography (PET) study, a melody generation task in amateur musicians has been shown to activate the prefrontal cortex (PFC; right BA 44 and left BA 45), motor areas [premotor cortex (PMC), supplementary motor area (SMA), and pre-SMA], bilateral superior temporal gyrus (STG), insula, basal ganglia, thalamus, and cerebellum (Brown et al., 2006). Blood oxygenation level dependent (BOLD) functional magnetic resonance imaging (fMRI) studies have shown the following results: tasks with the anticipation of sound sequences activate the PFC, premotor areas, and frontostriatal system (Leaver et al., 2009). A similar task that involved the expectancy of a musical ending complex has been shown to activate the superior frontal gyrus (SFG), middle frontal gyrus (MFG), inferior frontal gyrus (IFG), SMA, superior parietal lobule (SPL), STG, and striatum (Seger et al., 2013). In opera singers, singing activates the primary sensorimotor areas and basal ganglia (Kleber et al., 2010). Whereas different cortical areas are activated by different dimensions of musical information processing, such that the dorsal visual stream is activated by melodic processing, and the ventral visual stream is activated by rhythmic processing (Bengtsson and Ullén, 2006), the cortical motor areas (the PMC, SMA, and pre-SMA), basal ganglia, and cerebellum are commonly activated by many music-related tasks.

In general, acquiring skilled movements is accomplished through procedural learning, in which the basal ganglia play a central role (Graybiel, 2005). The basal ganglia are involved in the formation of motor skills, and several studies have demonstrated the activation of these areas during procedural motor learning (Graybiel, 2005). For example, an fMRI study has revealed that the sequence learning of finger movements activates the putamen and prefrontal and motor cortical areas (Lehéricy et al., 2005). Similar brain regions are activated during learning sound sequences (Leaver et al., 2009). The dorsal striatum, which consists of the caudate nucleus and putamen, receives convergent glutamatergic afferents from a large part of the cerebral cortex and functions as the gateway to the basal ganglia (Graybiel, 2005; Kreitzer and Malenka, 2008; Nagano-Saito et al., 2014). The signals in the basal ganglia are subjected to modulation by dopaminergic afferents from the substantia nigra pars compacta and ventral tegmental area. The major target of the nigrostriatal dopaminergic afferents is the dorsal striatum, and this system is particularly involved in motor control and cognition (Alexander et al., 1986). The mesolimbic dopaminergic afferents project to the ventral striatum (Graybiel, 2005). This pathway constitutes a major part of the reward system and is activated in association with reward expectancy (Alexander et al., 1986). Dopamine-mediated signals modulate corticostriatal synapses, thus forming the central part of the actor-critic model for reinforcement learning (Joel et al., 2002; Montague et al., 2004). The signals processed in the striatum are returned to cortical areas through the striato-pallido-thalamo-cortical pathway (Alexander et al., 1986). The striatum is thought to provide instructions to the cortex (Graybiel, 2005), because learning-related changes occur earlier in the striatum than in the PFC of monkeys performing reversals in a conditional association task (Pasupathy and Miller, 2005). Reinforcement learning of how to play a musical instrument requires the integration of multisensory attention, cognitive control, and outcome expectations, which is a striking property of striatal function (Horvitz, 2002). Therefore, the architecture of a network that connects these systems with different domains and converges in the striatum should be critical in developing a musical performance capability.

Current brain network analysis techniques based on graph theory can reveal the network properties of the entire brain (Cole et al., 2010; Van Den Heuvel and Sporns, 2011). A graph is composed of nodes and edges or connections between nodes. Nodes represent brain regions or voxels. There are several graph metrics, including degree and betweenness centrality, that characterize the network properties of nodes and global network properties (Rubinov and Sporns, 2010). Graph theoretical analysis suggests that the putamen is one of the brain network nodes that has significantly high degrees (i.e., the number of connections with other nodes; Van Den Heuvel and Sporns, 2011). Other similar nodes include the SFG, SPL, and precuneus. These nodes are included in the cognitive control network or the default mode network. These networks have often been reported to be negatively correlated with each other and are characterized as having high global connectivity (Cole et al., 2010), which probably reflects the demands for collecting or integrating various types of information to accomplish their tasks. Among the subcortical structures, the amygdala, hippocampus, putamen, and thalamus have been demonstrated to have high global connectivity (Cole et al., 2010). Several nodes have high degrees, constituting a rich-club organization (Van Den Heuvel and Sporns, 2011, 2013). A network with rich-club organization contains hubs that are more densely connected among themselves than with nodes of lower degrees (Van Den Heuvel and Sporns, 2011). A similar network organization has been found by an analysis of structural networks using diffusion tensor imaging data (Van Den Heuvel and Sporns, 2011). The analysis identified 12 strongly interconnected bihemispheric hub regions, including the SFG, SPL, precuneus, hippocampus, putamen, and thalamus. These hub regions were more densely interconnected than was expected solely on the basis of their degrees; therefore, these regions were identified as a rich-club. Such hub regions receive massive inputs from various areas and process a high load of information. An advantage of a network with rich-club organization is the ability to efficiently distribute the load of highly demanding information processing across the hubs. A functional connectivity analysis to determine the voxel-wise distributions of striatum connectivity has revealed a wide distribution across cortical areas, suggesting converging connectivity (Di Martino et al., 2008; Barnes et al., 2010; Choi et al., 2012). This converging corticostriatal connectivity has been confirmed both structurally and functionally (Jarbo and Verstynen, 2015).

A study using voxel-based morphometry (VBM) has reported that, compared with nondancers, professional female ballet dancers have decreased gray matter volumes in the left PMC, SMA, putamen, and SFG as well as decreased white matter volumes in both corticospinal tracts, both internal capsules, the corpus callosum, and the left anterior cingulum (Hänggi et al., 2010). Another VBM study has found that professional pianists have smaller gray matter volumes in the bilateral striatum, compared with amateur pianists or nonmusicians (James et al., 2014). Our VBM study has shown a decreased gray matter volume in the right caudate nucleus of the musicians’ brains compared with that in the brains of nonmusicians (Sato et al., 2015). All the subjects were right-handed females. Musicians have greater volumes in other regions, for example, in the right IFG, left middle occipital gyrus (MOG), bilateral lingual gyrus (Sato et al., 2015), left IFG, right mid orbital gyrus, left intraparietal gyrus, right fusiform gyrus, and left Heschl’s gyrus (HG; James et al., 2014). Therefore, the decreased volumes in the motor cortical areas and striatum of musicians and ballet dancers contrast with the increased volumes in other cortical areas. In a study focusing on pianists, the striatum volume has been found to be highest in nonmusicians, intermediate in amateur pianists, and lowest in professional pianists (James et al., 2014). We have found the same tendency in our VBM study; the caudate volume decreases in the order of nonmusician, music hobbyist, and music expert (Sato et al., 2015). Therefore, the reduction in striatal volume is considered to be a consequence of the neuroplasticity induced by long-term training.

Musical training-related neuroplasticity induces also functional changes in the brain of musicians (Munte et al., 2002; Herholz and Zatorre, 2012; Barrett et al., 2013). For instance, pianists have been shown to exhibit weaker activations, during a bimanual sequential finger movement task, than nonmusicians within a network including the anterior cingulate cortex (ACC), right dorsal PMC, basal ganglia, and cerebellum (Haslinger et al., 2004). This result indicates that professional pianists recruit the motor network to a lesser degree than nonmusicians (Haslinger et al., 2004). In addition, musicians exhibit no significant differences in activation levels between parallel and mirror bimanual finger movements (Haslinger et al., 2004). Parallel finger movements are more challenging for nonmusicians, and therefore, increased activation has been detected in nonmusicians. Musicians may lack significantly increased activation because they would have developed a network for high-level motor skills. Therefore, the functional network for skilled movement control in musicians would differ from that in nonmusicians. There are parallel cortico-striato-pallido-thalamo-cortical (CSPTC) loops composed of the motor, oculomotor, prefrontal, orbitofrontal, and cingulate circuits (Alexander et al., 1986). Motor skills are acquired through the modification of the information flow in the motor CSPTC circuit (Graybiel, 2005). Because the dorsal striatum is a critical node of the CSPTC circuit, receiving convergent input from a large part of the cerebral cortex, and functions as the gateway to the basal ganglia, we hypothesized that long-term musical training alters the functional connectivity of the dorsal striatum. To test this hypothesis, we analyzed the functional connectivity of the striatum (caudate/putamen) in musicians and nonmusicians. All of the participants were female. This study sought to examine how the functional connectivity of the striatum differs between female musicians and nonmusicians.

## Materials and Methods

### Participants

This study was approved by the Sophia University and Juntendo University Ethics Committees. University students majoring in music (*n* = 26; age: 18−27 years; mean = 21.5 years) and nonmusic disciplines (*n* = 26; age: 19−27 years; mean = 21.3 years) were recruited for this study using advertisements. All of the participants were healthy, right-handed Japanese females and had no history of neurological or neuropsychiatric diseases. The students majoring in music began musical training at 3–5 years of age, and their training continued to the start of the present study. All of the subjects specialized in classical music and played various instruments (piano, violin, cello, contrabass, clarinet, or trumpet). The nonmusic disciplines included philosophy, literature, linguistics, economics, law, psychology, science, and engineering. All participants provided written informed consent before the study commenced.

### Image Acquisition and Preprocessing

Neuroimaging was performed using the Philips Achieva 3.0 Tesla MRI scanner at the Juntendo University Hospital. Resting-state fMRI were acquired using a T2*-weighted gradient-echo echo-planar imaging (EPI) pulse sequence with TE = 30 ms and TR = 2000 ms. The field of view (FOV) was 240 × 240 mm (64 × 64 matrix) and the flip angle was 90°. The number of axial slices was 33 with no gap. The voxel size was 3.75 × 3.75 × 4.00 mm. The resting state session consisted of 200 scans (6 min 40 s). During the session, participants were instructed to relax but keep awake with their eyes closed. The first four volumes were discarded, and the remaining 196 volumes were preprocessed using the CONN toolbox (Whitfield-Gabrieli and Nieto-Castanon, 2012) running on MATLAB (Version 8.3.0, The MathWorks Inc., 2014). The fMRI data were first realigned and subsequently normalized to the standard Montreal Neurological Institute (MNI) template as implemented in the statistical parametric mapping (SPM) software platform. The slice timing was corrected according to the slice order, which was interleaved ascending. All functional images were spatially smoothed using a Gaussian filter kernel (FWHM = 8 mm). Image artifacts originating from head movement were handled using the ART scrubbing procedure.^1^ Signal contributions from white brain matter, cerebrospinal fluid, and micro head-movement (six parameters) were regressed out from the data. After the regression, fMRI data was bandpass filtered (0.008–0.09 Hz), linear detrended, and despiked.

### Analyses

Both graph theory analyses and functional connectivity were performed using the CONN toolbox. First, we explored the functional connectivity of the striatum in detail to examine how the connectivity was altered in musicians. In individual analysis, Pearson’s correlation coefficients were calculated between the seed time course and the time courses of all other voxels, which provided a seed-to-voxel connectivity matrix. Positive and negative correlation coefficients defined positive and negative functional connectivity, respectively (Whitfield-Gabrieli and Nieto-Castanon, 2012). The correlation coefficients were then converted to normally distributed scores using Fisher’s transform, which were subsequently used in the population-level analysis. The connectivity matrix with converted scores was compared between the music and nonmusic groups. The significance testing was done by controlling the false discovery rate (FDR) at *p* < 0.05. Next, we performed graph theory analysis to see the differences in graph measures. For each subject, the residual BOLD time courses were extracted from 132 regions of interest (ROIs) that cover the whole brain and the ROI-to-ROI correlation matrix was calculated from the time courses. The correlation coefficients were converted to normally distributed scores using Fisher’s transform. Thus obtained ROI-to-ROI matrix was thresholded at 0.2 to construct a binary connectivity matrix, from which graph measures were calculated. The measures of interest were global efficiency, local efficiency, betweenness centrality, cost, average path length, clustering coefficient, and degree or the number of connections for each node (ROI) to all other nodes (Rubinov and Sporns, 2010). Between-group differences in the graph measures were statistically tested.

## Results

**Figure 1:**
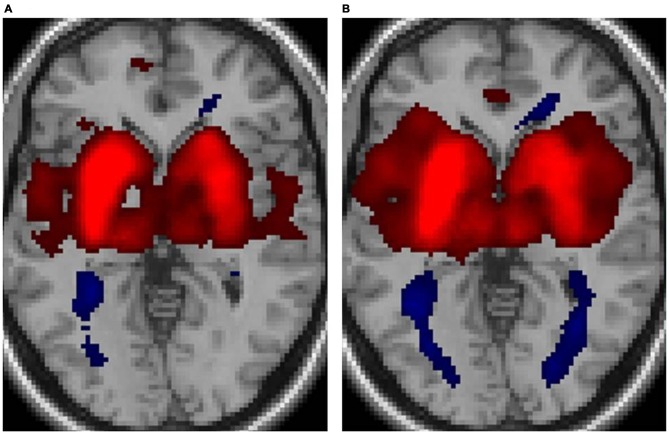
**Functional connectivity of the putamen for (A) musicians and (B) nonmusicians.** Both positive (warm colors) and negative (cool colors) connectivities are represented (*p* < 0.05, false discovery rate (FDR)).

**Figure 2:**
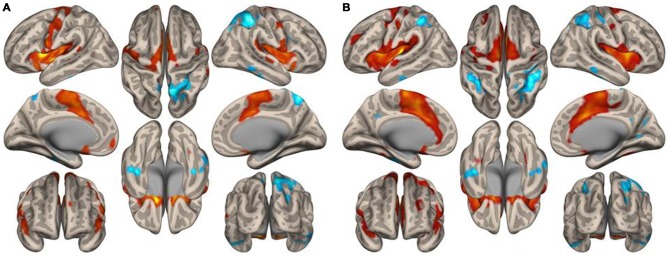
**Surface maps of the functional connectivity of the left putamen seed for (A) musicians and (B) nonmusicians.** Both positive (warm colors) and negative (cool colors) connectivities are represented (*p* < 0.05, FDR).

Figure 1 displays the functional connectivity profiles of the putamen in both groups. For both positive and negative functional connectivities, the profile was wider in nonmusicians than in musicians. Figure 2 shows the surface maps of the functional connectivity of the left putamen in the musicians and nonmusicians. The nonmusicians had wider functional connectivity profiles in the opercular/insular regions, SMA, and cingulate gyrus. In contrast, the musicians seemed to have developed a wider functional connectivity profile in the precentral gyrus; however, the strength of the functional connectivity did not differ significantly between the groups. The differences in the functional connectivities between the two groups are summarized in Table 1. The musicians had significantly decreased functional connectivity between the left putamen and the bilateral frontal operculum (FO), followed by the cerebellum (vermis 7) and the left insular cortex (IC). The functional connectivity between the right putamen and the right FO, left IC, and ACC was decreased in the musicians.

**Table 1:** Functional connectivity of the putamen and caudate nucleus: musicians vs. nonmusicians (*p < 0.05, FDR).

Seed	Target	*t* value	*p* (uncorrected)
Putamen.L	FO.R	−4.56	0.0000*
	FO.L	−3.96	0.0002*
	Vermis7	−3.30	0.0018
	IC.L	−2.97	0.0045
	ACC	−2.91	0.0053
	IC.R	−2.81	0.0070
Putamen.R	FO.R	−3.40	0.0013
	IC.L	−3.09	0.0032
	ACC	−2.95	0.0048
	IC.R	−2.89	0.0057
	CO.L	−2.86	0.0061
Caudate.L	Cblm6.R	−4.23	0.0001*
	Cblm6.L	−3.67	0.0006*
	Cblm8.R	−3.48	0.0011*
	Cblm8.L	−2.91	0.0054
	Cblm3.L	−2.73	0.0088
	aSMG.L	−2.70	0.0094
Caudate.R	Cblm8.R	−2.68	0.0099

*CO, central operculum; Cblm, cerebellum; aSMG, anterior supramarginal gyrus*.

**Figure 3:**
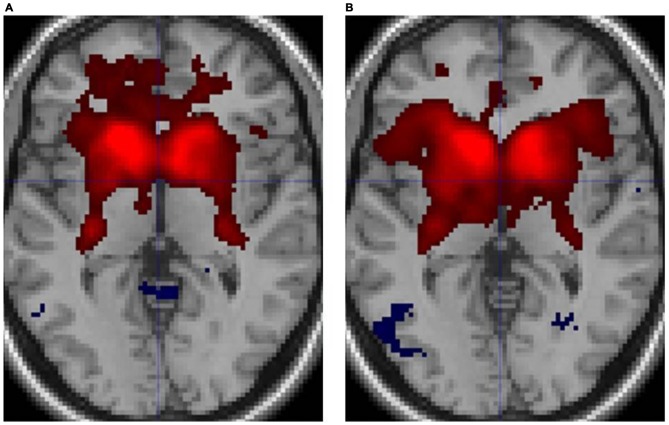
**Functional connectivity of the caudate nucleus in (A) musicians and (B) nonmusicians.** Both positive (warm colors) and negative (cool colors) connectivities are represented (*p* < 0.05, FDR).

**Figure 4:**
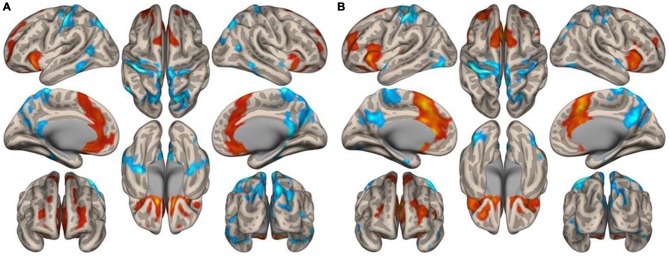
**Surface maps of the functional connectivity of the left caudate nucleus seed for (A) musicians and (B) nonmusicians.** Both positive (warm colors) and negative (cool colors) connectivities are represented (*p* < 0.05, FDR).

Figure 3 illustrates the functional connectivity profiles of the caudate nucleus in both groups. In the musicians, the functional connectivity profile was less extended laterally but extended more to the frontal regions. Figure 4 displays surface maps of the functional connectivity of the left caudate nucleus in the musicians and nonmusicians. In contrast to the results for the putamen, a positive functional connectivity with the precentral gyrus was not observed in either group. However, the left caudate nucleus had negative functional connectivity with the pre- and postcentral gyri in both groups. The functional connectivity profiles in the FO/anterior insula (AI) regions, SMA, and ACC were more extended in the nonmusicians, whereas the functional connectivity profile in the ventral medial prefrontal cortex (mPFC) was wider in the musicians. There were extended negative functional connectivity profiles in and around the precuneus, posterior cingulate cortex (PCC), and retrosplenial cortex; these connectivities were not observed in the functional connectivity of the putamen. The differences in the functional connectivities between the two groups are summarized in Table 1. The musicians had significantly decreased functional connectivity between the left caudate nucleus and the cerebellar regions (6LR and 8L).

**Figure 5:**
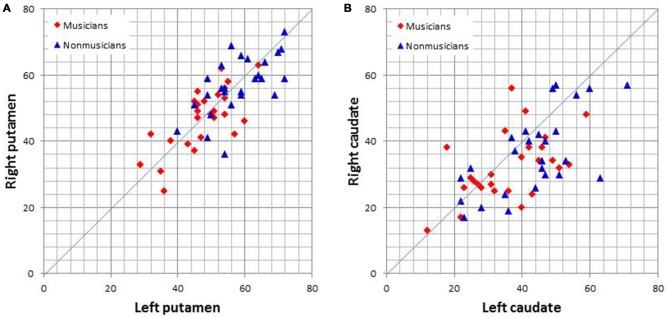
**Scatter plots of the degrees (i.e., the number of connections) of the left and right putamen (A) and caudate nucleus (B)**.

Graph theoretical analysis of the entire brain functional network revealed that musicians had significantly lower global efficiency, cost, and degree and a higher average path length in the bilateral putamen (*p* < 0.05, FDR). Table 2 lists the degrees of the putamen and caudate nucleus. The bilateral putamen, but not the caudate nucleus, had significantly lower degrees or fewer connections in musicians than in nonmusicians. Figure 5 shows the degrees of the left and right putamen and caudate nuclei. The degrees did not differ between the left and right putamen in musicians (*p* = 0.791) and nonmusicians (*p* = 0.672). Nonmusicians had a higher degree in the left caudate nucleus than in the right caudate nucleus (*p* = 0.047), whereas this tendency was weaker at an insignificant level in musicians (*p* = 0.190).

**Table 2:** **Degrees (i.e., the numbers of connections) of putamen and caudate nucleus in musicians and nonmusicians**.

		Musician		Nonmusician	
		Mean	SD	Mean	SD	*t* value	*p* (FDR)
Putamen	L	47.58	8.45	58.15	8.76	−4.42	0.007
Putamen	R	46.92	9.26	57.12	8.85	−4.07	0.011
Caudate	L	36.15	11.64	43.35	12.65	−2.13	0.504
Caudate	R	32.15	9.98	36.31	12.25	−1.38	0.693

## Discussion

### Corticostriatal Connectivity

The functional connectivity analysis has revealed the functional connectivity profiles between the cortical areas and the striatum. In both groups, there was a marked difference in the functional connectivity of the corticostriatal network between the caudate nucleus and the putamen. Specifically, the caudate nucleus connected with the SFG and MFG, whereas the putamen connected with more caudal areas including the precentral gyrus, results consistent with those from previous studies (Di Martino et al., 2008; Barnes et al., 2010; Choi et al., 2012). Similarly, the caudate nucleus connected with the FO/AI, whereas the putamen connected with the remaining caudal part of the opercular/insular regions. The same trend was observed in the medial cortex; the connected region of the caudate nucleus extended rostrally to the mPFC, whereas the connected region of the putamen shifted caudally. These tendencies were preserved in both groups but were more pronounced in the musicians than in the nonmusicians. The putamen connected with the precentral and motor areas, SMA, opercular/insular regions, and ACC in both groups. Consistently with the graph theoretical analysis in this study, the music group exhibited decreased connectivity of the putamen with the ACC and bilateral FO/AI. The significant decrease in the connectivity between the putamen and bilateral FO and the decrease in the connectivity between the putamen and ACC in the musicians are intriguing because the anterior (frontal) part of the cingulo-opercular network (CON) has been suggested to play a role in task-general tonic alertness as a fundamental function (Sadaghiani and D’esposito, 2015). The decreased connectivity between the putamen and CON in musicians may indicate that musical performance requires the deallocation of tonic alertness to an external environment. Although this interpretation seems counterintuitive, musical performance requires concentration on performance or introspection. The negative connectivity of the putamen and caudate nucleus with regions in the parietal cortex in both groups is also interesting because the parietal cortex is a principal component of the network for attentional and cognitive control of various kinds of information including visuospatial information and episodic memory (Cabeza et al., 2008; de Graaf et al., 2010; Nelson et al., 2010; Rosen et al., 2015). These issues are to be investigated in a future study using appropriate tasks.

### Network Properties

Our graph theoretical analysis demonstrated that the female musicians had a significantly decreased degree of the bilateral putamen compared with the nonmusicians, whereas the degree of the caudate nucleus was only modestly decreased in the musicians. Complex movements, including both sequential ordering and rhythm, are represented widely across cortical areas (Kornysheva and Diedrichsen, 2014; Bednark et al., 2015). In the entire brain network, the putamen is one of the nodes with high degrees of connections (Van Den Heuvel and Sporns, 2011). The result that the degrees of the bilateral putamen were significantly decreased in musicians suggests more selective convergence of information from cortical areas to the putamen in musicians. Regarding the laterality of connections, the putamen did not show laterality in both musicians and nonmusicians whereas the caudate nucleus in nonmusicians, but not in musicians, showed a higher number of connections in the left compared with the right caudate nucleus. A graph theoretical analysis has suggested that patients with Tourette syndrome have a network characterized by a shorter path length, a higher number of functional connections, stronger functional connections, and a loss of hubs (Worbe et al., 2012). The authors of the related study have proposed that this pattern of functional changes in the corticostriatal networks in patients may reflect a defect in brain maturation. The patients in the study also lost flexibility and exhibited redundant expressions in bodily movements. Pianists with musician’s dystonia, a movement disorder, also have a larger gray matter volume in the right middle putamen, compared with healthy pianists (Granert et al., 2011). This larger volume has been found to be associated with the presence of task-specific hand dystonia, whereas a smaller volume reflects the skill of piano playing. Thus, it appears that musical training reshapes the corticostriatal network such that the connectivity is more selective with a smaller striatum. The cerebellum is also involved in motor skill learning (Dayan and Cohen, 2011). A previous study has observed significantly larger cerebellar volumes in male keyboard players, but detected no significant difference in female keyboard players (Hutchinson et al., 2003). Other studies have found greater cerebellar volumes in musicians than in nonmusicians (Gaser and Schlaug, 2003; Han et al., 2009; Abdul-Kareem et al., 2011). However, a recent study has reported that musicians have smaller cerebellar volumes than nonmusicians (Baer et al., 2015). The authors of the study have found that better timing performance, a longer musical experience, and beginning musical training at an earlier age are negatively correlated with regional cerebellar volumes. The putamen is the principal node in the striatum of the CSPTC motor circuit (Alexander et al., 1986). Therefore, the finding that musicians had a less extended network of the putamen would be indicative that musical training modifies this motor circuit. This finding has not been reported previously, to the best of the authors’ knowledge.

### Limitations

When we recruited the participants using advertisements, music students who applied for the participation were all female. Then, we restricted controls to female students majoring in nonmusic disciplines. It is therefore uncertain whether the result in this study is also applicable to male students. In previous studies reporting smaller striatal volumes (Hänggi et al., 2010; James et al., 2014), the ballet dancers were all female (Hänggi et al., 2010) while the pianists were mixed with males and females (James et al., 2014). Because the smaller corticostriatal network is thought to be a consequence of selective pruning through long-term musical training, it would be improbable that this result depends on the gender of participants. However, this issue is to be studied.

## Conclusion

Musical performance requires complex information processing, including the integration of relevant information represented widely across cortical areas. The putamen, which has a high degree of connection among nodes of the entire brain, can function as a hub for the integration of such information. However, this corticostriatal network is smaller or selective in female musicians than in nonmusicians, consistently with results from previous anatomical studies reporting reduced volumes of the dorsal striatum in musicians and ballet dancers. The authors consider that long-term musical training constructs such a network by pruning unnecessary connections to optimize performance.

## Author Contributions

ST and EK planned and conducted all the experiments. ST analyzed the data and wrote the manuscript.

## Conflict of Interest Statement

The authors declare that the research was conducted in the absence of any commercial or financial relationships that could be construed as a potential conflict of interest.

## Footnotes

^1^www.nitrc.org/projects/artifact_detect/
